# Dual-Tracer PET-MRI-Derived Imaging Biomarkers for Prediction of Clinically Significant Prostate Cancer

**DOI:** 10.3390/curroncol30020129

**Published:** 2023-01-30

**Authors:** Bernhard Grubmüller, Nicolai A. Huebner, Sazan Rasul, Paola Clauser, Nina Pötsch, Karl Hermann Grubmüller, Marcus Hacker, Sabrina Hartenbach, Shahrokh F. Shariat, Markus Hartenbach, Pascal Baltzer

**Affiliations:** 1 Department of Urology, Medical University of Vienna, 1090 Vienna, Austria; 2 Department of Urology and Andrology, University Hospital Krems, 3500 Krems, Austria; 3 Karl Landsteiner University of Health Sciences, 3500 Krems, Austria; 4 Working Group of Diagnostic Imaging in Urology, Austrian Society of Urology, 1090 Vienna, Austria; 5 Department of Biomedical Imaging and Image Guided Therapy, Division of Nuclear Medicine, Medical University of Vienna, 1090 Vienna, Austria; 6 Department of Biomedical Imaging and Image Guided Therapy, Division of General and Pediatric Radiology, Medical University of Vienna, 1090 Vienna, Austria; 7 HistoConsultingHartenbach, 89081 Ulm, Germany; 8 Comprehensive Cancer Center, Medical University of Vienna, 1090 Vienna, Austria; 9 Department of Urology, Weill Medical College of Cornell University, New York, NY 10021, USA; 10 Department of Urology, University of Texas Southwestern, Dallas, TX 75390, USA; 11 Department of Urology, Second Faculty of Medicine, Charles University, 116 36 Prague, Czech Republic; 12 Hourani Center for Applied Scientific Research, Al-Ahliyya Amman University, Amman 19328, Jordan; 13 Karl Landsteiner Institute of Urology and Andrology, 1010 Vienna, Austria

**Keywords:** prostate cancer, PET/MRI, imaging biomarkers, dual tracer

## Abstract

Purpose: To investigate if imaging biomarkers derived from 3-Tesla dual-tracer [(18)F]fluoromethylcholine (FMC) and [^68^Ga]Ga-PSMA^HBED-CC^ conjugate 11 (PSMA)-positron emission tomography can adequately predict clinically significant prostate cancer (csPC). Methods: We assessed 77 biopsy-proven PC patients who underwent 3T dual-tracer PET/mpMRI followed by radical prostatectomy (RP) between 2014 and 2017. We performed a retrospective lesion-based analysis of all cancer foci and compared it to whole-mount histopathology of the RP specimen. The primary aim was to investigate the pretherapeutic role of the imaging biomarkers FMC- and PSMA-maximum standardized uptake values (SUVmax) for the prediction of csPC and to compare it to the mpMRI-methods and PI-RADS score. Results: Overall, we identified 104 cancer foci, 69 were clinically significant (66.3%) and 35 were clinically insignificant (33.7%). We found that the combined FMC+PSMA SUVmax were the only significant parameters (*p* < 0.001 and *p* = 0.049) for the prediction of csPC. ROC analysis showed an AUC for the prediction of csPC of 0.695 for PI-RADS scoring (95% CI 0.591 to 0.786), 0.792 for FMC SUVmax (95% CI 0.696 to 0.869), 0.852 for FMC+PSMA SUVmax (95% CI 0.764 to 0.917), and 0.852 for the multivariable CHAID model (95% CI 0.763 to 0.916). Comparing the AUCs, we found that FMC+PSMA SUVmax and the multivariable model were significantly more accurate for the prediction of csPC compared to PI-RADS scoring (*p* = 0.0123, *p* = 0.0253, respectively). Conclusions: Combined FMC+PSMA SUVmax seems to be a reliable parameter for the prediction of csPC and might overcome the limitations of PI-RADS scoring. Further prospective studies are necessary to confirm these promising preliminary results.

## 1. Introduction

Prostate cancer (PC) is among the most frequent malignancies in European men and is responsible for a significant number of cancer related deaths [1]. Nevertheless, there is evidence that a specific number of diagnosed PCs, namely “clinically insignificant” PCs (defined as ISUP grade 1), will never develop any clinical symptoms [2,3,4]. Indeed, many studies have consistently shown high 10-year cancer specific survival (CSS) rates of more than 90% for well-differentiated ISUP grade 1 PCs [5,6]. There is, therefore, a clinical need for accurate differentiation between “clinically significant” (defined as ISUP grade 2 or higher) (csPC) and “clinically insignificant” PCs, inducing a fundamental change in traditional diagnostic approaches [7].

Multiparametric magnetic resonance imaging (mpMRI) has, over the last years, become the most accurate local staging modality in this regard, helping in the identification of csPC. Many studies have reported a high sensitivity and specificity for both the detection and localization of csPC compared to previously used diagnostic modalities including prostate specific antigen (PSA) kinetics and standard systematic prostate biopsy [8,9].

The probability of the detection of PC with MRI-identified lesions has been standardized using the Prostate Imaging Reporting and Data System (PI-RADS) score, which has been recently updated to improve its reproducibility [10]. A published meta-analysis including 13 studies with suspected PC patients showed that the positive predictive value (PPV) for csPC with PI-RADS scores of 3, 4, and 5 were 12%, 48%, and 72%, respectively, with a high heterogeneity among the included studies [11]. This suggests that csPC can be missed with this technology due to MRI-invisible cancers, the reader’s misinterpretation, and possibly technical issues during biopsy [12]. 

Molecular imaging and the use of specific target probes such as [(18)F]fluoromethylcholine (FMC) positron emission tomography (PET) and [^68^Ga]Ga-PSMA^HBED-CC^ conjugate 11 (PSMA)-PET promise to overcome these limitations [13,14]. In this regard, combined hybrid imaging using FMC-PET/mpMRI and PSMA-PET/mpMRI achieved very high sensitivities for detecting csPC in previously published studies, improving the diagnostic accuracy and pretherapeutic assessment of PC compared to both PET and mpMRI alone [13,14,15]. However, these studies were limited by their sample size, study design, and pathologic evaluation.

Therefore, our aim was to investigate if imaging biomarkers derived from the 3T dual-tracer (FMC and PSMA) PET/mpMRI can adequately predict csPC. We investigated the feasibility of pretherapeutic combined FMC- and PSMA-PET/mpMRI as the local PC staging modality and compared imaging biomarkers derived from FMC- and PSMA-PET to the mpMRI parameters and the PI-RADS score. 

## 2. Material and Methods

### 2.1. Patients

This study was a retrospective analysis embedded in a prospective diagnostic trial (clinicaltrials.gov NCT02659527) including 77 consecutive patients with biopsy proven PC, who had undergone 3T dual-tracer (FMC and PSMA) PET/mpMRI followed by robotic-assisted radical prostatectomy (RP) between April 2014 and July 2017. Inclusion criteria of the aforementioned study were patients with clinical suspicion for localized prostate cancer, based on a PSA above 4.0 ng/mL and total to free PSA ratio above 22%, and/or two consecutive rising PSA values. Key exclusion criteria were previous therapy with androgen deprivation, recent prostate biopsy within 21 days, insufficient pathologic report of biopsy, or intolerance to used radiotracers. We performed a retrospective lesion-based analysis of all cancer foci within the investigated patient group and compared it to the whole-mount histopathologic RP specimen. All patients were treated with RP according to the recommendations of the guidelines. All surgical specimens were processed according to the standard pathologic procedures, staged with the AJCC TNM classification, and graded with the WHO/ISUP 2014 grading system [3] by a dedicated uro-pathologist. The primary aim of the study was to investigate the pretherapeutic role of imaging biomarkers derived from 3T dual-tracer PET/MRI (FMC- and PSMA-maximum standardized uptake values (SUVmax)) for the prediction of csPC and to compare it to the mpMRI methods (T2w, DCE, ADC) and PI-RADS score. All investigations were conducted in accordance with the Declaration of Helsinki and national regulations. The study was approved by the Ethics Committee (permit 1985/2014) and the drug authorities (EudraCT: 2014-004758-33). 

### 2.2. Imaging Protocol and Analyses

All PET-MRI examinations were performed using a hybrid PET-MRI system (Biograph mMR, Siemens, Germany) capable of simultaneous data acquisition. The system consists of an MRI-compatible state-of-the-art PET detector integrated in a 3.0-T whole-body MRI scanner. In short, the PET detector technology relies on lutetium oxyorthosilicate scintillation crystals in combination with MRI-compatible avalanche photodiodes instead of photomultiplier tubes. The PET component uses a 3-dimensional (3D) acquisition technique and offers an axial FOV of approximately 23 cm and a transversal FOV of 45 cm. The gradient system of the MRI scanner operates with a maximum gradient strength of 45 mT/m and a slew rate of 200 T/m/s in all three axes.

Patients received dual tracer PET/MRI scans, starting with a local static 5 min emission scan with 3 MBq/kg body weight FMC and a 45 min local list mode scan immediately after the injection of 2 MBq/kg body weight PSMA intravenously. Pelvic PET acquisition in the case of FMC was started 45 min post injection of the radiotracer. While acquiring the prostate MRI sequences, PSMA was injected dynamically and acquired simultaneously in the prostate/pelvic region for 45 min using listmode acquisition. 

The review of the PET/MRI images was performed separately by two experienced certified nuclear medicine physicians together with an experienced radiologist using Hermes Hybrid 3D (Hermes Medical Solutions Stockholm), while the assessment of MR images to assign PI-RADS v2.1 scores was conducted using AGFA IMPAXX EE software.

### 2.3. Follow-Up

Follow-up consisted of the standard follow-up after RP. In general, patients underwent physical examination and PSA testing every 3 months for the first two years, every 6 months from the second to the fifth year, then yearly.

### 2.4. Statistical Analyses

The primary objective of this study was the diagnostic accuracy of dual-tracer PET/MRI for csPC defined as an ISUP grade of 2 or above. Descriptive statistics of the cohort were performed. Receiver operating characteristics (ROC) analysis was used to quantify the univariable diagnostic performance using the area under the ROC curve (AUC) metric. Multivariable feature combination was modeled using the exhaustive Chi squared interaction detection (CHAID) algorithm. Minimum node size was set to 5, a minimum *p*-value of Bonferroni corrected 0.05 was used. Ordinally ordered terminal node categories were used to measure the multivariable model AUC in ROC analysis. The DeLong test was used to compare the AUCs, a *p*-value of 0.05 was considered significant. Statistical evaluation was carried out using STATA (version 14StataCorp, College Station, Texas, United States)).

## 3. Results

### 3.1. Patients

Overall, 77 patients met the inclusion criteria and were included in the study. The clinicopathologic features of patients and tumors after 3T dual-tracer PET/mpMRI followed by robotic-assisted RP are shown in Table 1. Median age was 70 at the time of RP with a median PSA of 8.1 ng/mL. Overall, 9.1%, 19.5%, 36.3%, 14.3%, and 20.8% of patients had ISUP 1, 2, 3, 4, and 5 at the time of RP, respectively. Of all the 104 cancer foci, 69 were clinically significant (66.3%) and 35 were clinically insignificant (33.7%). The median FMC SUVmax was 5 (4–6.9) and the median FMC+PSMA SUVmax was 14.3 (11.1–20.6) MBq in all of the cancer foci.

### 3.2. Prediction of Clinically Significant Prostate Cancer

**Figure 1:**
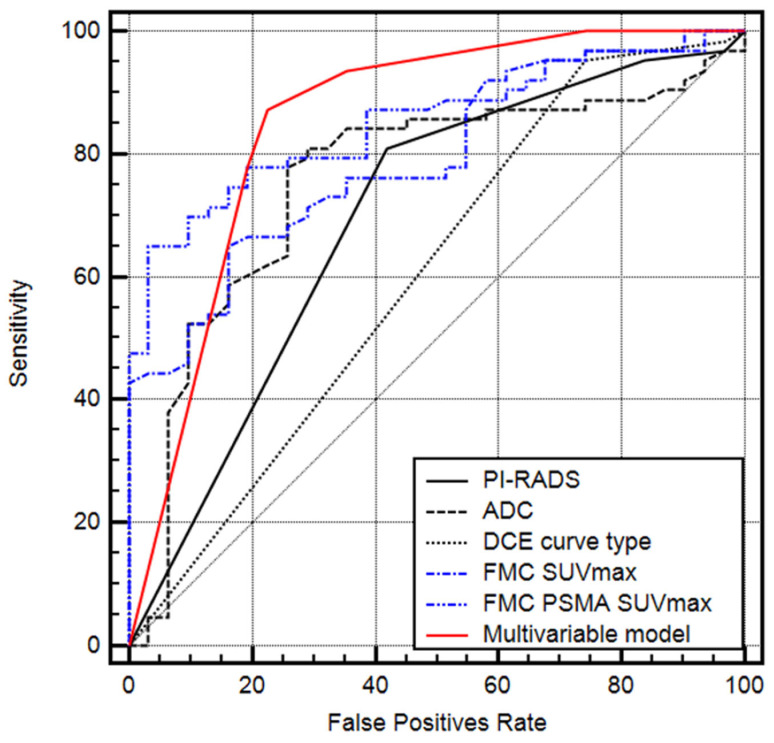
Receiver operating characteristic (ROC) analysis for the single parameters and the multivariable CHAID model.

The classification and regression tree methodology including PI-RADS score, apparent diffusion coefficient (ADC), dynamic contrast enhanced imaging (DCE) curve type, FMC SUVmax, FMC+PSMA SUVmax, lesion size, and zonal location as parameters revealed that FMC SUVmax and combined FMC+PSMA SUVmax were the only significant independent contributing parameters for the prediction of csPC (*p* < 0.001 and *p* = 0.049). ROC analysis showed that the AUC for the prediction of csPC was 0.695 [standard error (SE) 0.061] for the PI-RADS score (95% CI 0.591 to 0.786), 0.755 (SE 0.055) for the ADC (95% CI 0.656 to 0.838), 0.604 (SE 0.065) for the DCE curve type (95% CI 0.498 to 0.703), 0.792 (SE 0.795) for FMC SUVmax (95% CI 0.696 to 0.869), 0.852 (SE 0.038) for FMC+PSMA SUVmax (95% CI 0.764 to 0.917), and 0.852 (SE 0.038) for the multivariable CHAID model (95% CI 0.763 to 0.916) (Figure 1). Comparing different AUCs, the FMC+PSMA SUVmax and the multivariable model were more accurate for the prediction of csPC compared to the PI-RADS score (*p* = 0.0123 and *p* = 0.0253, respectively). A rule-out csPCa criterion was exclusively present in the multivariable model, correctly identifying 10/35 (28.6%) of all non-csPCa cases while missing no csPCa.

## 4. Discussion

There were two main findings to our study. First, we found that dual-tracer molecular imaging using FMC and PSMA was better at predicting the presence of csPC than conventional mpMRI graded according to the PI-RADS classification. Second, the multivariable model showed the possibility of a rule-out criterion for csPC that correctly identified 28.6% of all insignificant cancers by imaging, without missing a single case of csPC.

The introduction of mpMRI has greatly improved the detection of prostate cancer lesions [16]. The updated PI-RADS version 2 has subsequently made the grading of lesions more uniform, which has been expanded upon by the most recent update of the PI-RADS recommendations [10]. While the implementation of mpMRI followed by ultrasound fusion biopsy as a standard of care has significantly improved cancer detection and shown the potential to reduce overdiagnosis of insignificant PC, there is still a proportion of cancers being missed on mpMRI, and there are also suspicious lesions on imaging showing insignificant PC on biopsy and whole mount pathology. Several approaches have been evaluated to improve upon the accuracy of prostate cancer diagnosis, avoid unnecessary negative biopsies, and biopsies of insignificant PC. 

One of these was the evaluation of the quantitative parameters of prostate mpMRI in addition to the PI-RADS scoring system, which have previously been investigated. Polanec et al. showed that by measuring the minimum ADC-Map values in PI-RADS 4 and 5 lesions, unnecessary negative biopsy could be avoided in 33% of cases [17]. Chatterjee et al. published another study using the ADC-Map value, T2, and DCE enhancement rate as quantitative markers on a voxel by voxel basis. These quantitative risk-maps were matched to the RP specimen and could predict any cancer, csPC, defined as ISUP 2 or higher, and index lesions with an accuracy of 76.6%, 89.2%, and 100% [18]. These quantitative metrics have also been shown to be consistent over time and various scanners such as Wang et al. have published their recent results, showing good repeatability as well as reproducibility of the quantitative MRI parameters [19]. The addition of PET-based molecular imaging using different radiotracers has also shown the potential to improve upon the diagnostic accuracy of mpMRI alone. Berger et al. showed in a cohort of 50 patients undergoing RP with previous PSMA PET/MRI that with the addition of PSMA-PET, 100% of index lesions could be detected compared to 94% by MRI alone, and 93.5% vs. 51.6% of secondary cancer foci [20]. Similarly, Metser et al. also published their results, showing a significantly better diagnostic accuracy on the ROC analysis of PET/MRI vs. mpMRI (0.69 vs. 0.78; *p* = 0.04) in patients undergoing ^18^F-DCFPyL PET/mpMR for the suspicion of csPC, using ultrasound-guided fusion biopsy as the reference [21].

Other reliable diagnostic methods of further tumor characterization are generally based on tissue analysis, thus require an invasive procedure beforehand. These tissue-based biomarkers such as the Prolaris® or Decipher® can help identify and aid in clinical decision-making in patients with insignificant PC at high risk for upgrading and clinical progression, who should consider undergoing definitive treatment, or repeat biopsy as well as favorable intermediate risk patients at low risk of progression who are good candidates for active surveillance or conservative disease management [22,23]. 

Some studies have also shown a correlation between genomic classifiers and imaging characteristics, in some cases even using imaging to predict genomic markers. In Hectors et al.’s analysis of 64 patients, a radiomic model of 14 features showed correlation with the genomic signatures as well as good prediction of a Decipher® score above the threshold of 0.6 with an AUC of 0.84 [24]. However, data are scarce and conflicting, oftentimes not showing any synergistic effects of combining imaging and genomic biomarkers over each individual test alone [25]. Additionally, most of the available data were gathered retrospectively, allowing in some cases, the analysis of MRI-invisible PC lesions gnomically, but this would not be the case in clinical routine, as invisible lesions would not, or only by chance, be biopsied. For this reason, the simultaneous improvement in the detection as well as ideally non-invasive tumor classification would be of great benefit for patients and clinicians treating PC. 

In combining two molecular imaging tracers, knowing PSMAs but also to a degree, FMCs beneficial role in the detection of PC, we evaluated whether we could not only improve sensitivity, but further improve upon non-invasive tumor characterization. In our study, this was in fact the case, as the combined SUVmax of FMC and PSMA not only showed the best accuracy on the ROC analysis for the prediction of csPC, it also allowed for a rule-out criterion of csPC when implemented into a multivariable model, potentially allowing patients to forgo prostate biopsy, even in the case of suspicious lesions on mpMRI, and be directly entered into a program of surveillance, if proven in a larger prospective cohort. Additionally this has multiple potential uses during the follow-up of patients on active surveillance, as a confirmatory test in patients already diagnosed with low-risk PC as well as a form of longitudinal imaging follow-up in patients with elevated PSA and suspicious mpMRI. While this might seem to be very resource demanding, it is worth noting that tissue-based genomic biomarkers, which are becoming more widely available and used in patients on active surveillance, are within a very similar range of associated cost, as an instance of molecular imaging in many health care systems, while imaging retains the advantage of being a non-invasive diagnostic technique. Additionally, as multiple tracers can be applied simultaneously, the amount of time needed for such an examination is only increased marginally, depending on the tracers.

The combination of two radiotracers in molecular imaging has also shown proficiency, even in prospective studies, when guiding treatment decisions. Hofman et al. used the combination of PSMA and 2-flourine-18[18F]fluoro-2-deoxy-D-glucose (FDG) in their trial evaluating [^177^Lu]Lutetium-PSMA-617 as a therapy in advanced metastatic castration resistant PC (mCRPC) [26]. Patients with discordant results on imaging, showing FDG positive and PSMA negative lesions, or patients with very low PSMA expression were excluded from the study. This combination imaging was chosen due to the higher sensitivity, but it also allowed for tumor characterization. As described in another study, patients with discordant lesions represent a cohort of patients with very poor prognosis as these lesions tend to harbor de-differentiated PC cells, without PSMA expression, which are in most cases not suitable for radionuclide treatment and have grown resistant to most conventional PC therapies [27]. We chose the combination of PSMA and FMC in the setting of primary cancer, again, to improve the sensitivity, and allow for the identification of possible low risk cancers.

The biggest limitation of this study is its retrospective design. While the database was maintained prospectively as part of a prospective trial, this was a retrospective analysis. Furthermore, the cohort was already scheduled for RP, so it does not represent a normally distributed cohort of primary PC patients. Additionally, only patients with proven PC were included within this analysis, thus we cannot make any assumptions about the impact of dual-tracer PET/MRI in cancer detection overall or sensitivity in a biopsy naïve cohort, and we could only calculate the prediction of csPC on the RP specimen in patients with a previous positive biopsy. We did not perform any calculations on the survival outcomes such as PC recurrence, as this would not have been feasible with a cohort of this size and relatively short follow-up. Furthermore, the single center approach precludes robust estimates of the reproducibility and repeatability of the method. 

## 5. Conclusions

This study demonstrated that combined FMC+PSMA SUVmax in preoperative 3T dual-tracer PET/mpMRI seems to be a reliable parameter for the prediction of csPC and might overcome the limitations of MRI parameters and the interpretation according to the PI-RADS score. Further studies with bigger cohorts and a prospective randomized nature are necessary to confirm these promising but preliminary results.

## Figures and Tables

**Table 1:** Clinicopathologic features of 77 patients after 3T dual-tracer (FMC and PSMA) PET/mpMRI followed by robotic-assisted radical prostatectomy.

Age at RP (years), median (IQR)	70 (65–76)
PSA at RP (ng/mL), median (IQR)	8.1 (5.6–13.7)
Pathologic T staging after RP, *n* (%)	
2	41 (53.2)
3a	19 (24.7)
3b	17 (22.1)
Positive surgical margins, *n* (%)	24 (31.2)
ISUP grade on 77 RP specimen, *n* (%)	
1	7 (9.1)
2	15 (19.5)
3	28 (36.3)
4	11 (14.3)
5	16 (20.8)
Number of cancer foci in all 77 prostates, *n* (%)	104 (100)
Clinically insignificant cancer foci (ISUP 1)	35 (33.7)
Clinically significant cancer foci (≥ISUP 2),	69 (66.3)
Positive lymph nodes in histopathology, *n* (%)	14 (18.2)
Overall PI-RADS, *n* (%)	
3	2 (2.6)
4	16 (20.8)
5	59 (76.6)
FMC SUVmax of all cancer foci (MBq), median (IQR)	5 (4–6.9)
FMC + PSMA SUVmax of all cancer foci (MBq), median (IQR)	14.3 (11.1–20.6)

RP = radical prostatectomy, IQR = interquartile range, mpMRI = multiparametric magnetic resonance imaging, ISUP grade = International Society of Urological Pathology grade, FMC = fluoromethylcholine, PSMA = prostate specific membrane antigen, PI-RADS = Prostate Imaging Reporting and Data System, SUVmax = maximum standardized uptake value, MBq = megabecquerel.

## Data Availability

The complete dataset used for this analysis is available in a pseudonymized fashion upon request, and after acquisition of IRB approval as well as data sharing agreements between institutions.
